# High expression of GFAT1 predicts unfavorable prognosis in patients with hepatocellular carcinoma

**DOI:** 10.18632/oncotarget.15164

**Published:** 2017-02-07

**Authors:** Lili Li, Miaomiao Shao, Peike Peng, Caiting Yang, Shushu Song, Fangfang Duan, Dongwei Jia, Mingming Zhang, Junjie Zhao, Ran Zhao, Weicheng Wu, Lan Wang, Can Li, Hao Wu, Jie Zhang, Xin Wu, Yuanyuan Ruan, Jianxin Gu

**Affiliations:** ^1^ Key Laboratory of Glycoconjugate Research Ministry of Public Health, School of Basic Medical Sciences, Fudan University, Shanghai, P.R.China; ^2^ Department of Biochemistry and Molecular Biology, School of Basic Medical Sciences, Fudan University, Shanghai, P.R.China; ^3^ Institutes of Biomedical Sciences, Fudan University, Shanghai, P.R.China; ^4^ Department of General Surgery, Zhongshan Hospital, Fudan University, Shanghai, P.R.China; ^5^ Key Laboratory of Female Reproductive Endocrine Related Diseases, Obstetrics and Gynecology Hospital, Fudan University Shanghai, P.R.China

**Keywords:** GFAT1, hepatocellular carcinoma, overall survival, recurrence, prognostic factor

## Abstract

Hepatocellular carcinoma (HCC) is the second leading cause of cancer-related deaths worldwide. As a branch of glucose metabolism, hexosamine biosynthesis pathway (HBP) has been reported to play a critical role in the insulin resistance and progression of cancer. Glutamine:fructose-6-phosphate amidotransferase (GFAT) is the rate-limiting enzyme of the HBP; nevertheless, the prognostic value of GFAT1 in HCC remains elusive. In this study, we found that high expression of GFAT1 was significantly associated with serum alpha-fetoprotein (AFP), serum alanine aminotransferase (ALT), tumor size, tumor encapsulation, T stage and TNM stage. High GFAT1 expression was identified as an independent prognostic factor which predicted poor overall survival (OS) and recurrence-free survival (RFS) in HCC patients. Incorporation of GFAT1 expression could improve the prognostic accuracy of traditional TNM stage system. Integration of GFAT1 expression with other independent prognosticators generated a predictive nomogram, which showed better prognostic efficiency for OS and RFS in HCC patients. In vitro studies also revealed that GFAT1 promoted the proliferation, cell cycle progression, migration and invasion of HCC cells. In conclusion, GFAT1 is a potential prognostic biomarker for overall survival and recurrence-free survival of HCC patients after surgery.

## INTRODUCTION

Liver cancer is one of the most frequently diagnosed cancers, and hepatocellular carcinoma (HCC) occupies a large proportion (70% to 90%) of the primary liver cancers [1]. At early disease stages, surgical resection, liver transplantation, and ablation by radiofrequency or ethanol injection are conventional therapies, and survival at 5 years ranges between 50% and 70% [2]. Unfortunately, HCC is often diagnosed at an advanced/late stage when surgery is no longer applicable. To make it worse, high rate of postsurgical metastasis and relapse is a major challenge of HCC, owing to the fact that this disease is highly resistant to conventional chemotherapy and radiation [3]. Thus, better understanding the molecular basis can help us to find new target for the precise diagnosis and treatment of patients with HCC.

Metabolism is always aberrant in cancer cells compared to normal cells [4]. One of the most common phenomena is aberrant glucose metabolism. While most cellular glucose is metabolized by glycolysis, a minor branch (2–5%) of the glycolytic pathway shunted to the hexosamine biosynthesis pathway (HBP) [5]. GFAT is the first and the rate-limiting enzyme of the HBP, which catalyze fructose-6-phosphate (F-6-P) and glutamine to glucosamine-6-phosphate (GlcN-6-P) and glutamate. Subsequent steps converts GlcN-6-P to UDP-N-acetylglucosamine (UDP-GlcNAc), which is the monosaccharide donor for N-glycosylation or O-glycosylation. Among the three identified human GFAT isoforms, GFAT1 is the major form that is ubiquitously expressed [6–10]. Growing evidences demonstrate that aberrant glycosylation through HBP can modulate tumor malignant transformation in different cancers [11]. Recent study has shown that high GFAT1 expression was associated with worse progression-free survival and overall survival in triple-negative breast cancer [12]. However, as the rate-limiting enzyme of HBP, the role and the prognostic value of GFAT1 in patients with HCC has not been demonstrated.

In this study, we aimed to investigate the expression of GFAT1 in hepatocellular carcinoma and its relationship with clinicopathologic features and clinical outcome. Furthermore, a nomogram integrating GFAT1 expression and pathologic characteristics was established to predict the 3-year and 5-year overall survival and recurrence-free survival for the patients with HCC after surgery.

## RESULTS

### GFAT1 expression is decreased in HCC tissue samples

**Figure 1:**
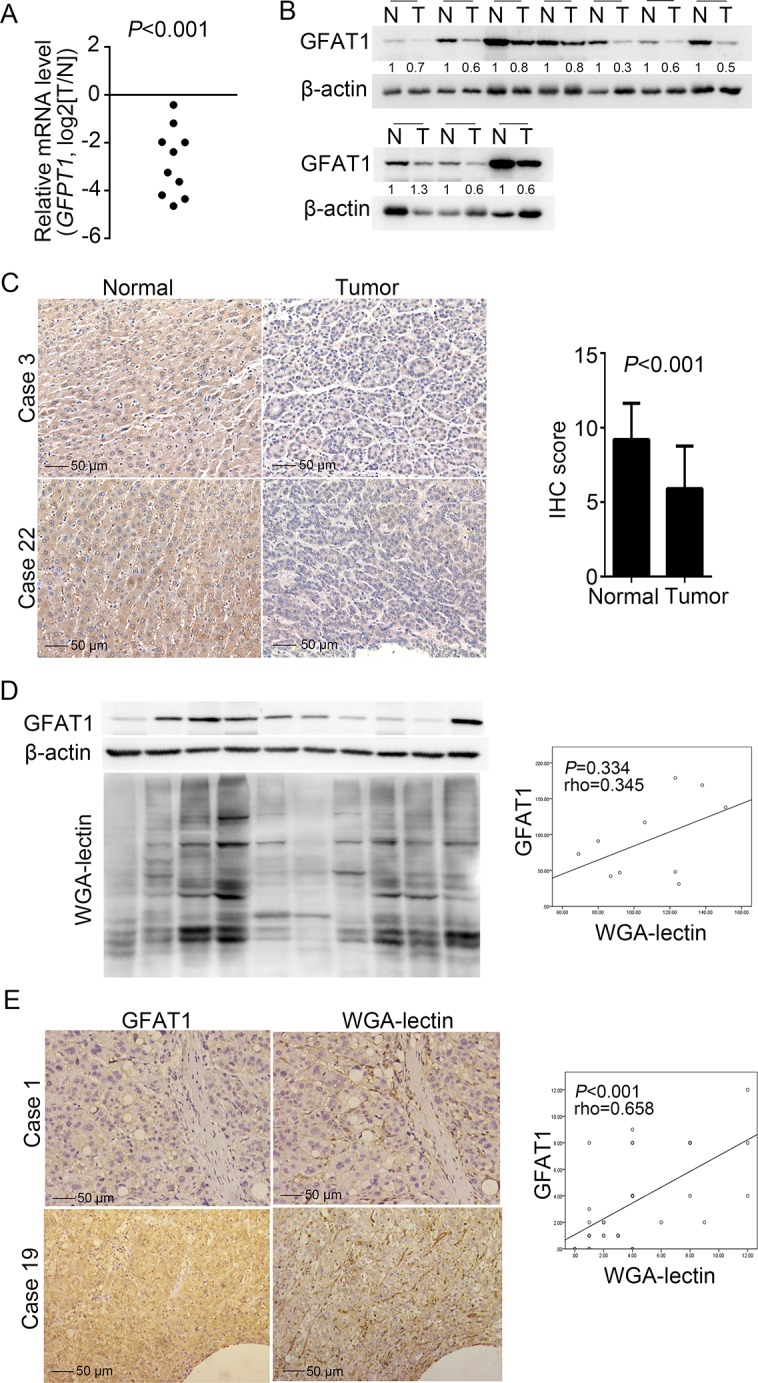
GFAT1 expression is decreased in HCC sample tissues (**A** and **B**) The expression of GFAT1 was examined by real-time PCR analysis (A) and western-blot (B) in 10 pairs of fresh HCC tissues and adjacent normal tissues. N, adjacent non-tumor sections; T, tumor sections. (**C**) The expression of GFAT1 in 40 pairs of HCC tissue sections and adjacent normal tissue sections was compared by IHC scoring. Images shown are representative results in 2 cases. (**D**) GFAT1 protein expression and WGA lectin staining were detected by western-blot in 10 fresh HCC tissues. (**E**) GFAT1 protein expression and WGA lectin staining in 40 HCC tissue sections were detected by IHC. Images shown are representative results in 2 cases. In (D) and (E), the correlation was analyzed by Spearman's ρ test. In (C) and (E), scale bar = 50 μm. *P* < 0.05 was considered statistically significant.

We first investigated the expression of GFAT1 in 10 paired fresh HCC tissues. Real-time PCR analysis revealed that the mRNA expression of GFAT1 was down-regulated in all HCC cases (Figure 1A). Western-blot also indicated that the GFAT1 protein levels were decreased in 9 out of 10 HCC cases (Figure 1B). In addition, immunohistochemistry (IHC) assay also indicated that the protein expression of GFAT1 was apparently lower in HCC tissues than that in adjacent normal tissues (Figure 1C). The GFAT1 expression was mainly localized in the cytoplasm of tumor cells (Figure 1C).

Since UDP-GlcNAc is a major end product of HBP and can provide N-acetylglucosamine for glycosylation, we also performed wheat germ agglutinin (WGA) lectin blot to determine the level of GlcNAcylation. In the 10 fresh HCC tissues, western blot analysis revealed that GFAT1 expression and WGA blot level were positively correlated but with no statistical significance (rho = 0.345, *P* = 0.334), possibly due to the limited sample size (Figure 1D). Meanwhile, in the 40 HCC sections, IHC assay confirmed the GFAT1 expression and WGA staining levels were positively and statistically significantly correlated (rho = 0.658, *P* < 0.001) (Figure 1E).

### Correlation between GFAT1 expression and clinicopathologic characteristics of HCC patients

**Figure 2:**
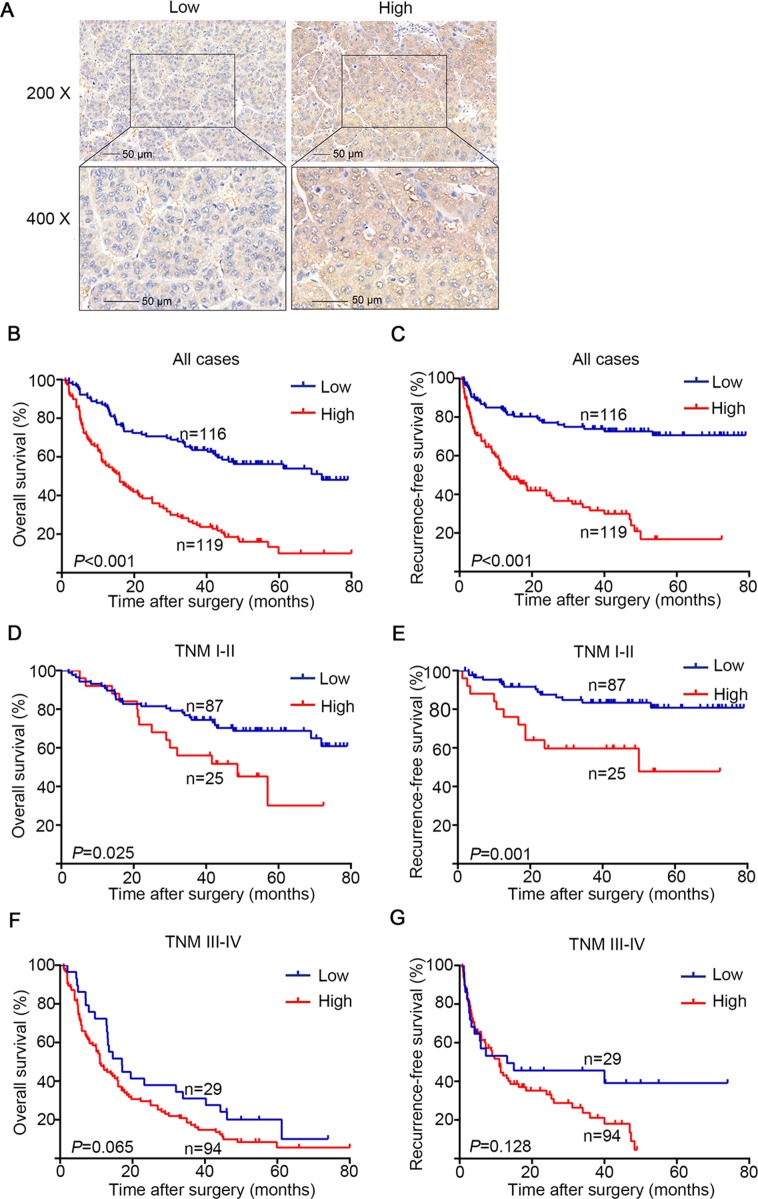
OS and RFS analysis of patients with hepatocellular carcinoma based on GFAT1 expression (**A**) Representative IHC images of GFAT1 low expression and GFAT1 high expression in HCC tissues. (**B**) Kaplan–Meier analysis of OS in all patients. (**C**) Kaplan–Meier analysis of RFS in all patients. (**D**) Kaplan–Meier analysis of OS in TNM I+II patients. (**E**) Kaplan–Meier analysis of RFS in TNM I+II patients. (**F**) Kaplan–Meier analysis of OS in TNM III+IV patients. (**G**) Kaplan–Meier analysis of RFS in TNM III+IV patients. *P*-value was calculated by log-rank test. In (A), scale bar = 50 μm.

To understand the clinicopathologic significance of GFAT1 in HCC, we next determined GFAT1 expression by IHC staining analysis in tissue microarray including 235 patients with HCC. The staining intensities were variable in tumor tissues (Figure 2A). For vast majority of HCC samples, GFAT1 expression was evenly scattered throughout the specimens in the majority of tumor tissues. Among the total 235 subjects, 116 (49.4%) patients were separated into the GFAT1 low expression subgroup and 119 (50.6%) patients were separated into the GFAT1 high expression subgroup according to the cut-off value.

The relationship between clinical pathological characteristics and GFAT1 expression was analyzed in Table 1. High expression of GFAT1 was positively associated with serum AFP (*P* < 0.001), serum ALT (*P* < 0.001), tumor size (*P* < 0.001), tumor encapsulation (*P* = 0.044), T stage (*P* < 0.001) and TNM stage (*P* < 0.001). GFAT1 expression was not relevant to other clinical characteristics in our study.

**Table 1:** Correlation between GFAT1 expression and clinicopathologic characteristics of patients with HCC

Characteristic	*N*	GFAT1 expression		*P*-value
		Low (*N* = 116)	High (*N* = 119)	
**Age (years)**				0.622
≤ 58	99	47 (47.5%)	52 (52.5%)	
> 58	136	69 (50.7%)	67 (49.3%)	
**gender**				0.652
Female	34	18 (52.9%)	16 (47.1%)	
Male	201	98 (48.8%)	103 (51.2%)	
**HbsAg**				0.921
Negative	48	24 (50.0%)	24 (50.0%)	
Positive	187	92 (49.2%)	95 (50.8%)	
**Serum AFP (ng/ml)**				< 0.001
≤ 20	84	58 (69.0%)	26 (31.0%)	
> 20	151	58 (38.4%)	93 (61.6%)	
**ALT (U/I)**				< 0.001
≤ 40	102	72 (70.6%)	30 (29.4)	
> 40	133	44 (33.1%)	89 (66.9%)	
**Liver cirrhosis**				0.005
No	66	23 (34.8%)	43 (65.2%)	
Yes	169	93 (55.0%)	76 (45.0%)	
**Tumor size (cm)**				< 0.001
≤ 5	108	70 (64.8%)	38 (35.2%)	
> 5	127	46 (36.2%)	81 (63.8%)	
**Tumor differentiation**				0.575
I-II	140	67 (47.9%)	73 (52.1%)	
III-IV	95	49 (51.6%)	46 (48.4%)	
**Tumor number**				< 0.001
single	185	104(56.2%)	81 (43.8%)	
multiple	50	12 (24.0%)	38 (76.0%)	
**Tumor capsule**				0.044
Absent	76	47 (61.8%)	29 (38.2%)	
Present	159	76 (47.8%)	83 (52.2%)	
**T stage**				< 0.001
T1+T2	116	73 (62.9%)	43 (37.1%)	
T3+T4	119	43 (36.1%)	76 (63.9%)	
**N stage**				0.672
N0	230	114(49.6%)	116 (50.4%)	
N1	5	2 (40.0%)	3 (60.0%)	
**M stage**				0.545
M0	225	112(49.8%)	113 (50.2%)	
M1	10	4 (40.0%)	6 (60.0%)	
**TNM stage**				< 0.001
I-II	112	87 (77.7%)	25 (22.3%)	
III-IV	123	29 (23.6%)	94 (76.4%)	

Abbreviations: HBsAg, hepatitis B virus surface antigen; AFP, a-fetoprotein; ALT, alanine aminotransferase; TNM, tumor node metastasis; GFAT, glutamine:fructose-6-phosphate amidotransferase. *P* < 0.05 is considered statistically significant.

### High GFAT1 expression was negatively correlated with OS and RFS of HCC patients

To further investigate the relationship between GFAT1 expression and HCC patients’ outcomes, Kaplan-Meier analysis was applied to evaluate the OS and RFS in the GFAT1 high expression and the GFAT1 low expression groups as mentioned above. The *P*-value was calculated by log-rank test. High expression of GFAT1 was found to be associated with poor OS (*P* < 0.001, Figure 2B) and RFS (*P* < 0.001, Figure 2C). To further investigate whether GFAT1 expression could stratify patients by different TNM stages, we divided the HCC patients into early-stage (TNM I–II) and advanced-stage (TNM III–IV) groups. In the early-stage subgroup, patients with high GFAT1 expression showed significantly shorter OS (*P* = 0.025, Figure 2D) and RFS (*P* = 0.001, Figure 2E). However, GFAT1 expression exhibited no statistically significant value in predicting the OS and RFS of HCC patients in the advanced-stage subgroup (Figure 2F and 2G), suggesting GFAT1 might be more valuable in predicting the outcome of HCC patients at early stage.

### GFAT1 expression is identified as an independent prognostic factor and could increase the predictive value of TNM stage

**Figure 3:**
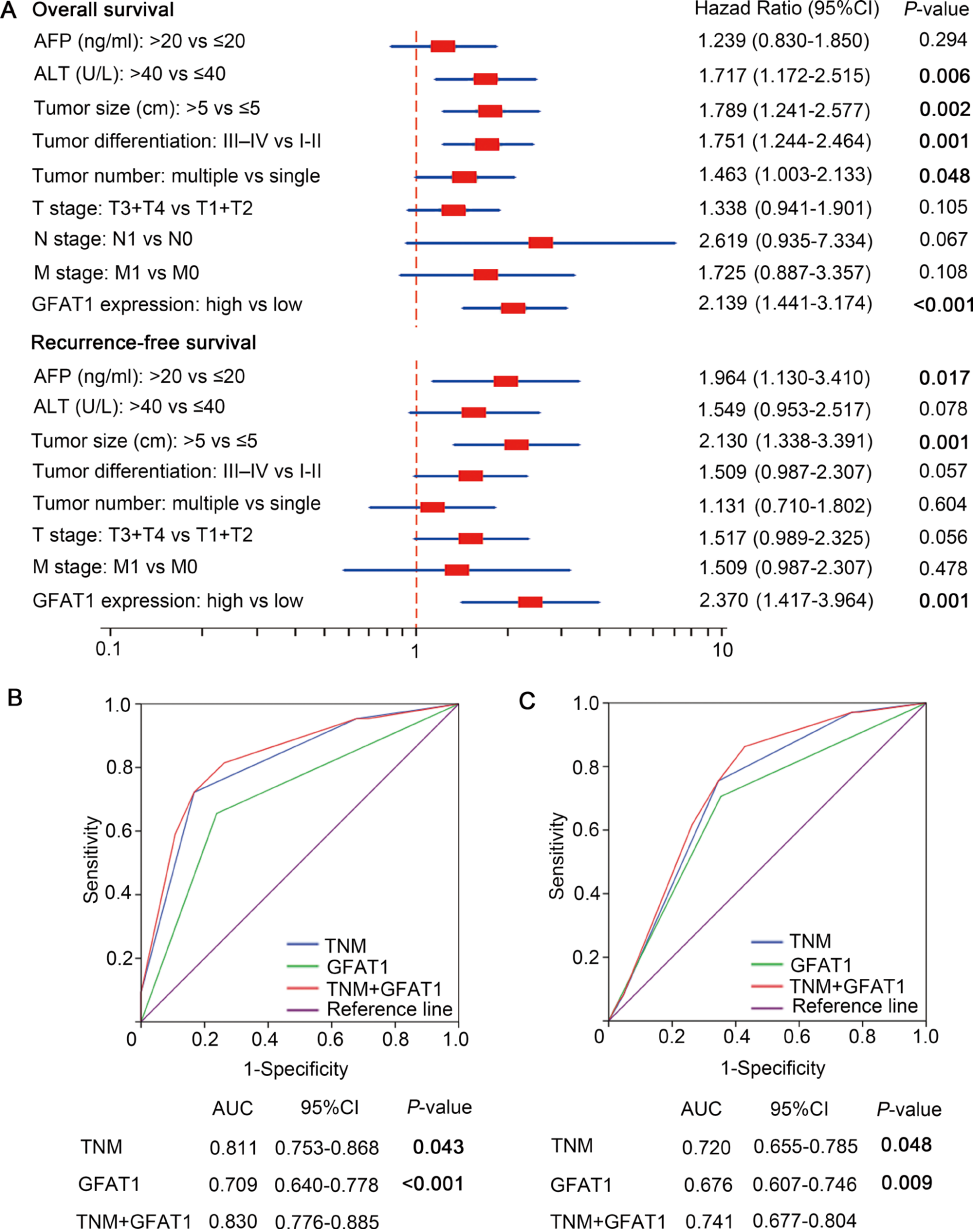
Multivariate Cox regression analysis and ROC analyses for predictive effect of GFAT1 (**A**) Independent prognostic factors were identified by Multivariate Cox analysis for OS and RFS. (**B** and **C**) ROC analysis of the sensitivity and specificity for the predictive value of TNM model, GFAT1 expression model and the combined TNM+GFAT1 model of OS (B) and RFS (C). *P* < 0.05 was considered statistically significant.

Univariate and multivariate analyses were performed to give a further analysis. As shown in Table 2, GFAT1 high expression group had a significantly increased risk of OS (HR, 2.995; 95% CI, 2.317 to 4.458, *P* < 0.001) and RFS (HR, 3.754; 95% CI, 2.674 to 5.926, *P* < 0.001). Those characteristics which were significant in the univariate analyses were incorporated into the multivariate analyses. We found that serum ALT (HR, 1.717; 95% CI, 1.172 to 2.515, *P* = 0.006), tumor size (HR, 1.789; 95% CI, 1.241 to 2.577, *P* = 0.002), tumor differentiation (HR, 1.751; 95% CI, 1.244 to 2.464, *P* = 0.001), tumor number (HR, 1.463; 95% CI, 1.003 to 2.133, *P* = 0.048) and GFAT1 expression (HR, 2.139; 95% CI, 1.441 to 3.174, *P* < 0.001) showed a significant risk in multivariate analyses and were determined as independent prognostic factors of OS (Figure 3A). Meanwhile, serum AFP (HR, 1.964; 95% CI, 1.130 to 3.410, *P* = 0.017), tumor size (HR, 2.130; 95% CI, 1.338 to 3.391, *P* = 0.001), and GFAT1 expression (HR, 2.370; 95% CI, 1.417 to 3.964, *P* = 0.001) were determined as independent prognostic factors of RFS (Figure 3A).

**Table 2:** Univariate Cox regression analysis of overall survival and recurrence-free survival

Characteristic	Overall survival		Recurrence-free survival	
	HR (95 % CI)	*P*-Value	HR (95 % CI)	*P*-Value
**Age (years)**				
≤ 58 *vs* > 58	1.354 (0.9131–1.920)	0.139	1.480 (0.912–2.228)	0.120
**gender**				
Male *vs* Female	1.236 (0.780–1.902)	0.386	1.002 (0.579–1.733)	0.992
**HbsAg**				
Positive *vs* Negative	1.194 (0.803–1.748)	0.394	1.080 (0.668–1.739)	0.756
**Serum AFP (ng/ml)**				
> 20 *vs* ≤ 20	1.983 (1.369–2.614)	< 0.001	2.672 (1.603–3.517)	< 0.001
**ALT (U/I)**				
> 40 *vs* ≤ 40	2.649 (1.904–3.611)	< 0.001	2.979 (1.948–4.270)	< 0.001
**Liver cirrhosis**				
No *vs* Yes	1.028 (0.720–1.467)	0.879	0.791 (0.508–1.202)	0.262
**Tumor size (cm)**				
> 5 *vs* ≤ 5	2.691 (1.967–3.743)	< 0.001	3.450 (2.293–5.018)	< 0.001
**Tumor differentiation**				
III–IV *vs* I-II	1.728 (1.289–2.535)	< 0.001	1.557 (1.064–2.432)	0.024
**Tumor number**				
Multiple *vs* Single	2.480 (2.204–5.438)	< 0.001	2.198 (1.642–4.826)	< 0.001
**Tumor capsule**				
Present *vs* Absent	1.177 (0.763–1.806)	0.467	1.007 (0.588–1.723)	0.979
**T stage**				
T3+T4 *vs* T1+T2	2.153 (1.588–3.026)	< 0.001	2.315 (1.593–3.496)	< 0.001
**N stage**				
N1 *vs* N0	2.648 (1.044–25.090)	0.044	1.633 (0.318–11.080)	0.487
**M stage**				
M1 *vs* M0	3.142 (2.703–23.700)	< 0.001	2.750 (1.463–20.360)	0.011
**TNM stage**				
III –IV *vs* I-II	4.447 (3.558–6.894)	< 0.001	5.417 (4.056–9.094)	< 0.001
**GFAT1 expression**				
High *vs* low	2.995 (2.317–4.458)	< 0.001	3.754 (2.674–5.926)	< 0.001

Abbreviations: HBsAg, hepatitis B virus surface antigen; AFP, a-fetoprotein; ALT, alanine aminotransferase; TNM, tumor node metastasis; GFAT, glutamine:fructose-6-phosphate amidotransferase. *P* < 0.05 is considered statistically significant.

We next incorporated the GFAT1 expression into the TNM stage to see whether GFAT1 expression could improve the prognostic accuracy of traditional TNM stage system in patients with HCC. According to the Receiver Operating Characteristic (ROC) analysis, we found that the combination of GFAT1 expression with TNM stage showed the higher predictive value (AUC 0.830; 95% CI 0.776 to 0.885) than TNM stage alone (AUC 0.811; 95% CI 0.753 to 0.868, *P* = 0.043) or GFAT1 expression alone (AUC 0.709; 95% CI 0.640 to 0.778, *P* < 0.001) for OS (Figure 3B). Similarly, incorporation of GFAT1 expression with TNM stage had better predictive value (AUC 0.741; 95%CI 0.677 to 0.804) than TNM stage alone (AUC 0.720; 95%CI 0.655 to 0.785, *P* = 0.048) or GFAT1 expression alone (AUC 0.676; 95%CI 0.607 to 0.746, *P* = 0.009) for RFS (Figure 3C). These results indicate that a combination of GFAT1 expression and TNM stage could generate a more accurate prognostic system.

### Predictive Nomogram for OS and RFS of HCC patients

**Figure 4:**
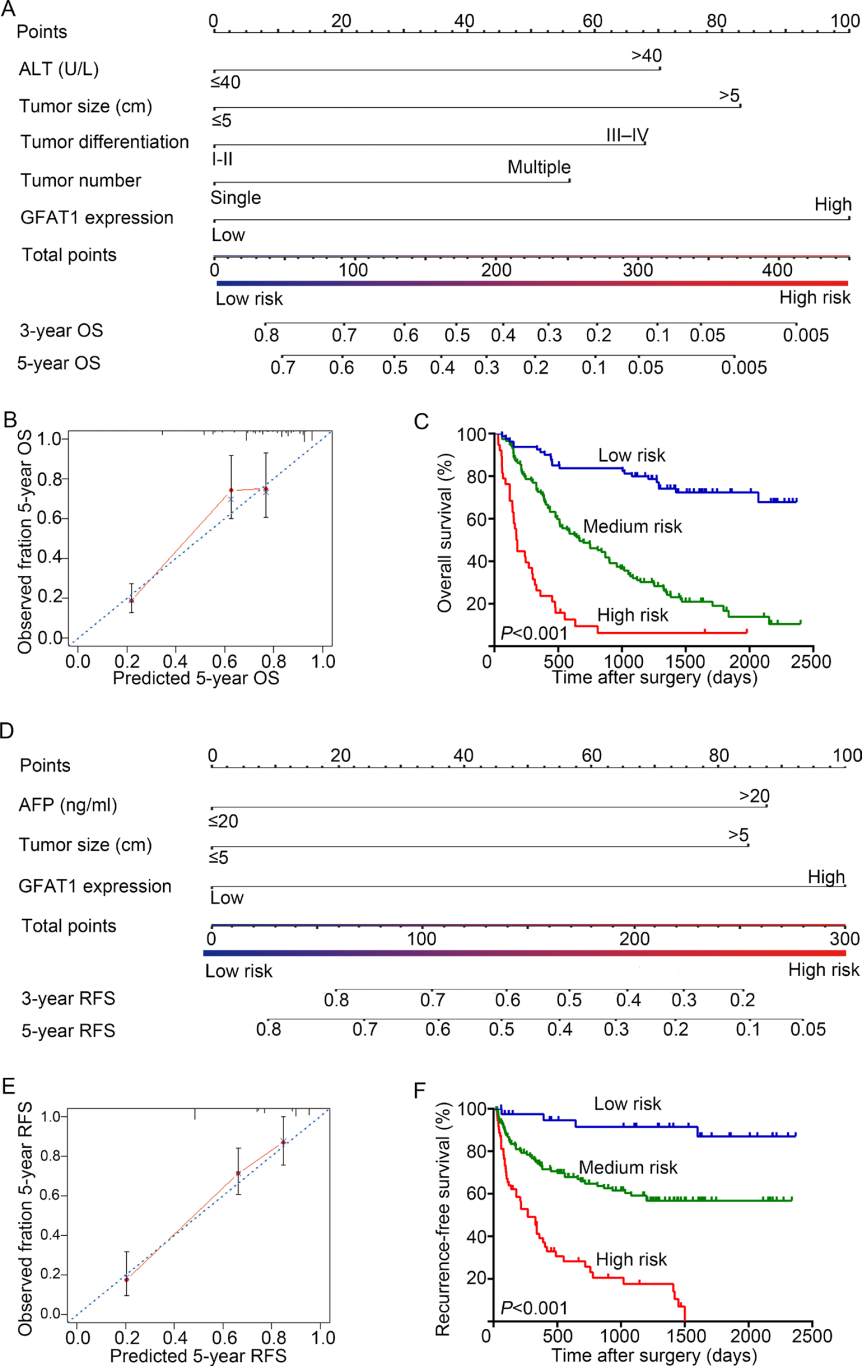
Nomogram for prediction of OS and RFS and Kaplan-Meier analysis of nomogram based model (**A** and **D**) Nomogram incorporated independent prognostic factors to predict overall survival (A) and recurrence-free survival (D) of patients with HCC. (**B** and **E**) The calibration plots for predicting 5 years OS (B) and RFS (E). The x-axis represents nomogram-predicted OS and RFS, the y-axis represents actual OS and RFS respectively. The dash line along the 45-degree indicated a perfect calibration in which the predicted probabilities are identical to the actual outcomes. (**C** and **F**) OS (C) and RFS (F) of patients were analyzed by Kaplan-Meier analysis according to the risk score of nomogram-based model.

A prognostic nomogram was constructed by integrating all these independent prognostic factors for OS and RFS. In the nomogram, each independent prognostic factor had a risk score and the total risk score was calculated by adding the risk score of different prognostic factors. Serum ALT, tumor size, tumor differentiation, tumor number, and GFAT1 expression were incorporated in the nomogram model for OS of the HCC patients (Figure 4A), while serum AFP, tumor size and GFAT1 expression were considered for RFS (Figure 4D). For internal validation, calibration curves for nomogram predicted 5-year overall survival rates and recurrence-free survival rates were built, respectively, and both curves showed an optimal agreement between actual results and the prediction by nomogram (Figure 4B and 4E). Based on the risk score, patients were stratified into three subgroups, including subgroups I for low risk score (< 25%), subgroup II for medium risk score (25%-75%) and subgroup III for high risk score (> 75%). OS (Figure 4C, *P* < 0.001) and RFS (Figure 4F, *P* < 0.001) in each group were found to increase following the trend from high- to low-risk groups, which demonstrated that scoring with the nomogram effectively discriminated the risk of postoperative survival in HCC patients.

### Overexpression of GFAT1 promotes the tumorigenicity of HCC cells *in vitro*

**Figure 5:**
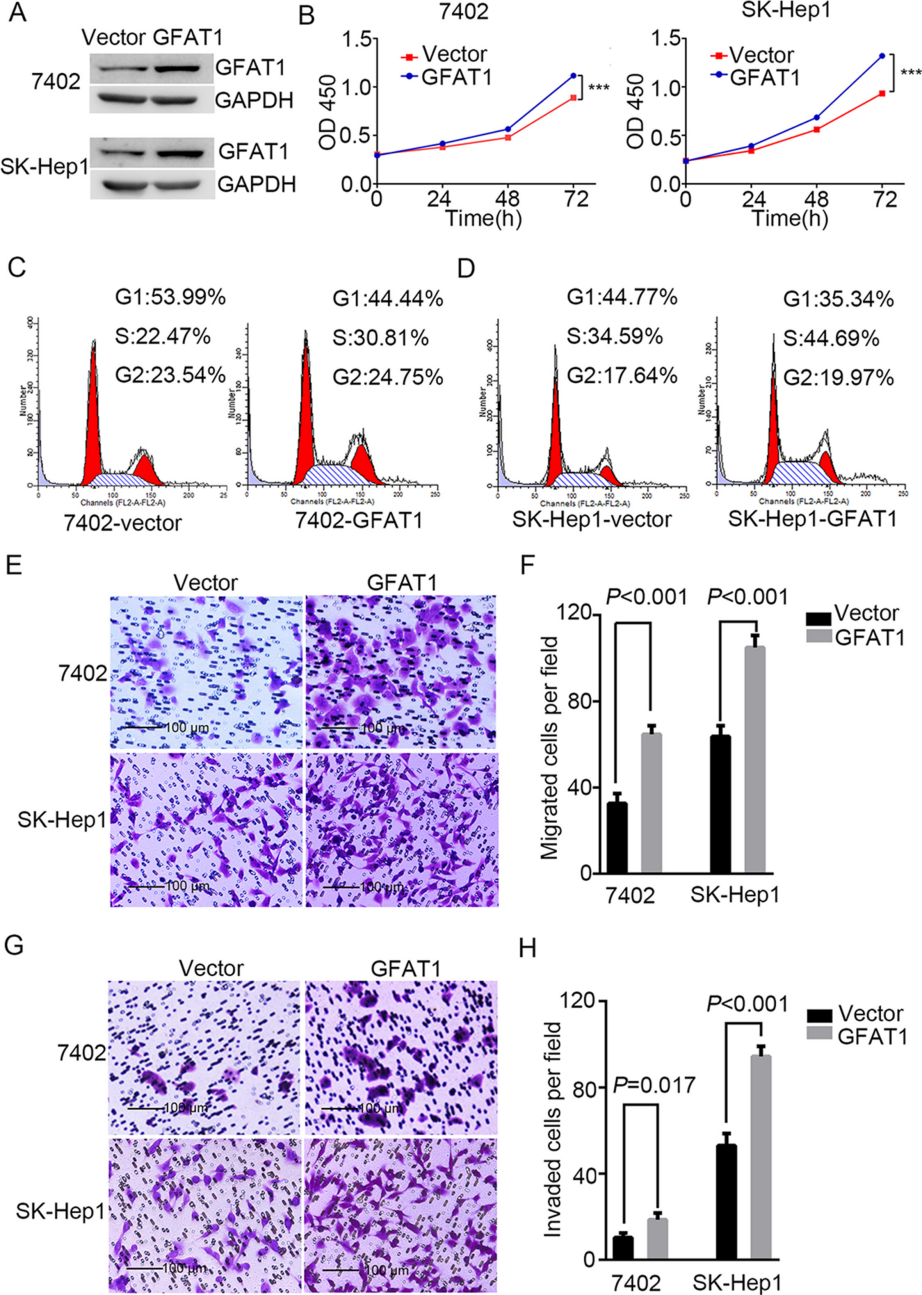
Overexpression of GFAT1 promotes the tumorigenicity of HCC cells *in vitro* (**A**) Western blot for efficiency of GFAT1 overexpression in BEL-7402 and SK-Hep1 cells. (**B**) CCK-8 assays for the effects of GFAT1 overexpression on the viability of HCC cells. (**C** and **D**) PI staining assays showing the effects of GFAT1 on cell cycle progression in BEL-7402 (C) and SK-Hep1 (D) cells. (E–G) Transwell assays for the effects of GFAT1 overexpression on migratory (**E** and **F**) and invasive (**G** and **H**) potentials of HCC cells. In (E) and (G), scale bar = 100 μm.

We next determined the effect of GAT1 overexpression on the tumorigenicity of HCC cell lines BEL-7402 and SK-Hep1 (Figure 5A). CCK8 assay revealed that GFAT1 promoted cell viability in both cell lines (Figure 5B). PI staining also indicated that overexpression of GFAT1 led to a significant increase in the percentage of cells at the S phase and a decrease in cells at the G1 phase (Figure 5C and 5D). Moreover, transwell analysis demonstrated that *in vitro* migration and invasion of HCC cells was significantly facilitated by overexpression of GFAT1 (Figure 5E–5H). Collectively, overexpression of GFAT1 could promote the tumorigenicity of HCC cells *in vitro*.

## DISCUSSION

It is well recognized that cancer cells are characterized by deregulated glucose metabolism. Compared to normal cells, cancer cells strongly upregulate glucose uptake and glycolysis in order to fuel cell growth and division and to provide increased yield of intermediate glycolytic metabolites as the substrates for the biosynthesis [13, 14]. The HBP is a relatively minor branch of the glycolytic pathway and functions as a cellular nutrient sensor. HBP is initiated by the first and rate limiting enzyme GFAT, which converts fructose-6-phosphate to glucosamine-6-phosphate [15]. Previous study has shown that glutamine analogs such as 6-diazo-5-oxo-L-norleucine (DON) and azaserine, the inhibitors of GFAT1, inhibited cancer cell growth [16], suggesting a potential role of GFAT in driving tumorigenesis.

Increased uptake of glucose is accompanied with increased flux into the HBP and the subsequent elevated glycosylation including *O*-GlcNAcylation, N-linked, and mucin type O-linked glycosylation. Therefore, GFAT1 plays a vital role in the cellular glycosylation reactions, and dysregulation of GFAT1 in HCC may result in aberrant glycosylation that contributes to tumor development. This is also confirmed in our study that the expression of GFAT1 was positively correlated with WGA lectin staining in HCC tissues (Figure 1D and 1E). Previous studies have also demonstrated that overexpression of GFAT1 in adipocytes could lead to increased glucose uptake and increased synthesis and storage of lipid, which caused the pathological hallmarks of diabetes, insulin resistance [17]. Therefore, HBP is considered as a potential target for type-2 diabetes treatment [18]. In addition, recent research have indicated that HBV infection could up-regulate the hexosamine biosynthesis pathway, and inhibition of HBP through GFAT1 can reduce HBV replication and expression [19]. Another study demonstrated that hexosamine biosynthesis pathway was elevated in CD133-positive subpopulation compared to CD133-negitive subpopulation in hepatocellular carcinoma [20], implying that HBP play a critical role in the maintenance of CSC-like phenotype. These studies indicate that GFAT1 may be a promising target for HCC prevention and treatment.

As far as I know, this is first study that proposes the clinical significance of GFAT1 expression in predicting the OS and RFS of patients with HCC. In addition, incorporation of GFAT1 could improve the prognostic efficiency of TNM stage. Nomogram is statistical model providing a more individualised prediction of prognosis based on a combination of variables and has been established in many cancers. In our study, nomogram incorporating the variables could well discriminate the risks for OS and RFS of HCC patients. However, we have to acknowledge that there are some limitations in our study. First, the expression of GFAT1 was detected by immunohistochemistry, which was a bit subjective. Second, the population enrolled in our study is relatively small, and a larger population should be employed to validate our conclusions. Third, our study was based on a retrospective analyses, a prospective study with more detailed clinicopathologic characteristics should be made to confirm our findings.

In conclusion, high GFAT1 expression is identified as an independent adverse prognostic factor that is associated with OS and RFS in HCC patients. In addition, a nomogram intergrating GFAT1 expression and other independent clinical factors can well discriminate the risks of the 3-year and 5-year OS and RFS. *In vitro* studies also indicate that overexpression of GFAT1 promotes tumorigenicity of HCC cells. Future studies may focus on the molecular mechanisms underlying the tumorigenic role of GFAT1 in HCC.

## MATERIALS AND METHODS

For tissue microarray detection, the tumor tissue samples from a total of 235 patients who underwent curative resection in Zhongshan Hospital, Fudan University in 2007 were collected. The clinical and pathological characteristics comprising age, gender, hepatitis B virus surface antigen (HBsAg), AFP, ALT, liver cirrhosis, tumor size, tumor differentiation, tumor number, tumor encapsulation and tumor stage were retrospectively collected. Tumor TNM stage were identified according to the American Joint Committee on Cancer 2010 TNM classification. The Edmondson grading system was used to grade tumor differentiation. OS was defined from the date of surgery to the day of death or the last follow up and RFS was calculated from the date of recurrence to the date of death or the last follow-up. Written informed consent on the use of clinical specimens from all subjects was obtained from all patients, and the use of clinical specimens was approved by the research medical ethics committee of Fudan University. The independent groups of 10 fresh paired HCC samples and 40 paraffin-embedded HCC sections were also collected at Zhongshan Hospital, Fudan University in Shanghai, China.

### Tissue microarray and immunohistochemistry

Tissue microarray (TMA) was constructed as described previously [21, 22]. Briefly, all the HCC tissues were histologically reviewed by HE staining and representative areas free from necrotic and haemorrhagic tissue were selected. Two cores of 1mm diameter were punched from each representative tumor tissues and the adjacent non-tumorous tissues respectively. Anti-GFAT1 antibodies (Cat# ab176775) was purchased from Abcam (Cambridge, MA, USA) and used for immunohistochemistry (IHC). The control slides were incubated in the absence of primary antibody to ensure the specificity of antibody. The staining intensity of each specimen was assessed by two independent pathologists blinded to the clinicopathological data. The staining intensity was scored as 0 (negative), 1 (weak), 2 (moderate) and 3 (strong). The distribution area (percentage of staining area of positive cells) was scored as 0 (< 5%), 1 (5%–25%), 2 (26%–50%), 3 (51%–75%) and 4 (> 75%). The immunoreactivity score ranged from 0 to 12 was derived by multiplying the staining intensities by distribution area. Finally, we defined 4 as the optimum cut-off value to dichotomize the patients into low and high groups according to the ROC curve analysis.

### Cell lines

HCC cell lines BEL-7402 and SK-Hep1 were obtained from Cell Bank of Type Culture Collection of Chinese Academy of Sciences, Shanghai Institute of Cell Biology, Chinese Academy of Sciences. Cells were cultured in RPMI 1640 or DMEM supplemented with 10% fetal bovine serum (FBS) at 37°C in a humidified atmosphere containing 5% CO2. All of the culture media were purchased from Sigma (St. Louis, MO, USA), and fetal bovine serum was purchased from Gibco (catalogue no. 16000-044; Grand Island, NY, USA).

### Real-time PCR

Total RNA was extracted from HCC tissues using TRIzol (Cat# 15596-026, Gibco BRL and Life Technologies) according to the manufacturer's instructions. Real-time PCR was performed as described in our previous report [23].

### Plasmids construction and transfection

The cDNA encoding human GFAT1 was obtained by PCR and was inserted into the pcDNA3.1 vector (Life Technologies, Carlsbad, CA, USA). Transfections were performed with Lipofectamine 3000 (Life Technologies, Carlsbad, CA, USA), according to the manufacturer's instructions.

### Statistical analysis

SPSS 22 (IBM Corporation, Armonk, NY, USA) was used to perform the statistical analyses. The relationship between GFAT1 expression and clinicopathologic variables were analyzed by χ2 test. Overall survival curves and recurrence-free survival curves were evaluated by Kaplan-Meier method and compared by Log-rank test. The Cox proportional hazards regression model was applied to evaluate multivariate analyses, and those statistically significant characteristics in univariate analysis were used to perform multivariate analysis. Nomogram was generated based on the results of multivariate analysis and by using the package of rms in R version 3.2.3. The prognostic accuracy of the models was evaluated by calibration plot. Receiver operating characteristic (ROC) analysis were applied to predict the accuracy of the clinical outcome by the parameters. Correlation of GFAT1 with WGA lectin levels was analyzed using nonparametric Spearman's ρ test. All statistical analyses were two-sided and *P* < 0.05 was regarded as statistically significant.
